# Supplementary figures and images for: Improvement of liver fibrosis, but not steatosis, after HCV eradication as assessment by MR-based imaging: Role of metabolic derangement and host genetic variants
Source: PLoS One. 2022 Jun 13;17(6):e0269641. doi: 10.1371/journal.pone.0269641 (PMC9191717; doi:10.1371/journal.pone.0269641)

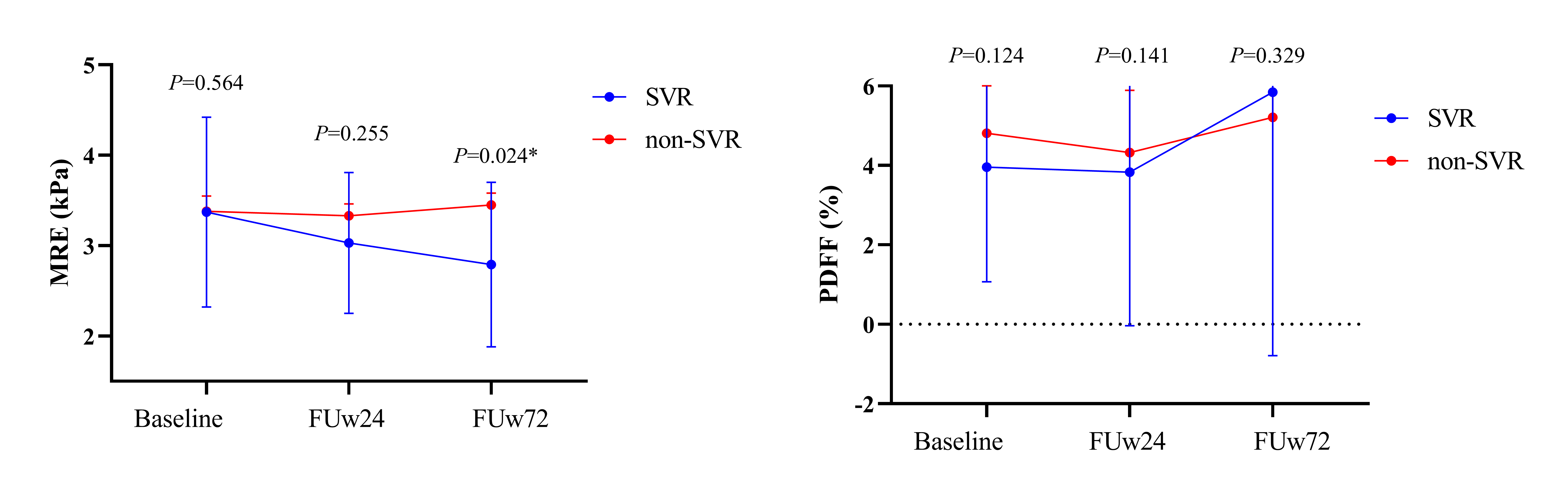

Supplement: S1 Fig — (TIF) [file pone.0269641.s001.tif]
